# Alternative Polyadenylation: a new frontier in post transcriptional regulation

**DOI:** 10.1186/s40364-020-00249-6

**Published:** 2020-11-25

**Authors:** Fanggang Ren, Na Zhang, Lan Zhang, Eric Miller, Jeffrey J. Pu

**Affiliations:** 1 Upstate Cancer Center, State University Of New York Upstate Medical University, Suite 331, CWB, 750 E. Adams Street, Syracuse, NY 13210 USA; 2 Laboratory of Hematology, the Second Hospital of Shanxi Medical University, Taiyuan, Shanxi China

## Abstract

Polyadenylation of pre-messenger RNA (pre-mRNA) specific sites and termination of their downstream transcriptions are signaled by unique sequence motif structures such as AAUAAA and its auxiliary elements. Alternative polyadenylation (APA) is an important post-transcriptional regulatory mechanism that processes RNA products depending on its 3′-untranslated region (3′-UTR) specific sequence signal. APA processing can generate several mRNA isoforms from a single gene, which may have different biological functions on their target gene. As a result, cellular genomic stability, proliferation capability, and transformation feasibility could all be affected. Furthermore, APA modulation regulates disease initiation and progression. APA status could potentially act as a biomarker for disease diagnosis, severity stratification, and prognosis forecast. While the advance of modern throughout technologies, such as next generation-sequencing (NGS) and single-cell sequencing techniques, have enriched our knowledge about APA, much of APA biological process is unknown and pending for further investigation. Herein, we review the current knowledge on APA and how its regulatory complex factors (CFI/IIm, CPSF, CSTF, and RBPs) work together to determine RNA splicing location, cell cycle velocity, microRNA processing, and oncogenesis regulation. We also discuss various APA experiment strategies and the future direction of APA research.

## Introduction

Splicing, capping, and polyadenylation are three major steps in processing pre-messenger RNA (pre-mRNA) to mRNA [1, 2]. Polyadenylation (poly(A)) involves in endonucleolytic cleavage of pre-mRNA and addition of the poly(A) tail at the cleavage site [1]. Individual pre-mRNA usually harbors a few cleavage/polyadenylation (C/P) sites (polyA sites or pA) [2]. Alternative polyadenylation (APA) can eventually produce several mRNA polyadenylation isoforms [3].

According to current understanding, APA is a comprehensive process accomplished via coordinative actions of several small molecules. The 3′-processing factors are the major targets of APA regulation [4]. Typical APA processing includes the following steps: (1) CFIm (cleavage factor I) binds to the UGUA field of pre-mRNA upstream of the pA site and attracts CPSF (cleavage and polyadenylation specificity factor) and CSTF (cleavage stimulation factor) to assemble at the end of RNA polymerase II; (2) as RNA polymerase II advances, CPSF binds to the pA signal sequence (e.g. AAUAAA) and CSTF is transferred to the new mRNA precursor, binding to the GU or U-rich sequence; (3) CPSF and CSTF initiate the cleavage of ~ 35 nucleosides after the pA signal sequence, and polyadenylation binding protein (PABPN1) in the nucleus will bind to the polyadenylation tail sequence to begin the PAP process; (4) while PAP-mediated polyadenylation continues, adenosine tails of~ 50–250 nucleotides (nt) are prepared (depending on the species of the organism) and CPSF dissociates from its binding sequence; (5) PABPN1 works as a molecular ruler during this APA progression, defining when the polyadenylation process should stop; (6) PAP begins to dissociate, although PABPN1 continues to maintain its binding status. The combination of above 6 steps in conjunction with the 5′-capping process promotes mRNA maturation and eventual exportation from nucleus to cytoplasm.

Approximately 50 ~ 80% of mammalian pre-mRNA transcripts have more than one pA sites [5, 6]. The 3′-UTRof mRNA harbors key RNA regulatory elements that determine when, where, and how much mRNA transcript will be translated [1]. APA is a crucial 3′-UTR post-transcriptional regulation mechanism. The 3′-UTR APA isoforms play various roles in determining mRNA stability, localization, half-life, and functions. Furthermore, previous studies demonstrated that APA is involved in disease progression and drug sensitivity, especially for drugs targeting chromatin modifiers [2, 7–9]. Though APA research is still in its early stage, its unique post-transcriptional regulatory effect makes it potentially both a biomarker for cancer prognosis and diagnosis, and a target for novel target therapy development [10, 11].

### How APA modulates pre-mRNA

**Fig. 1:**
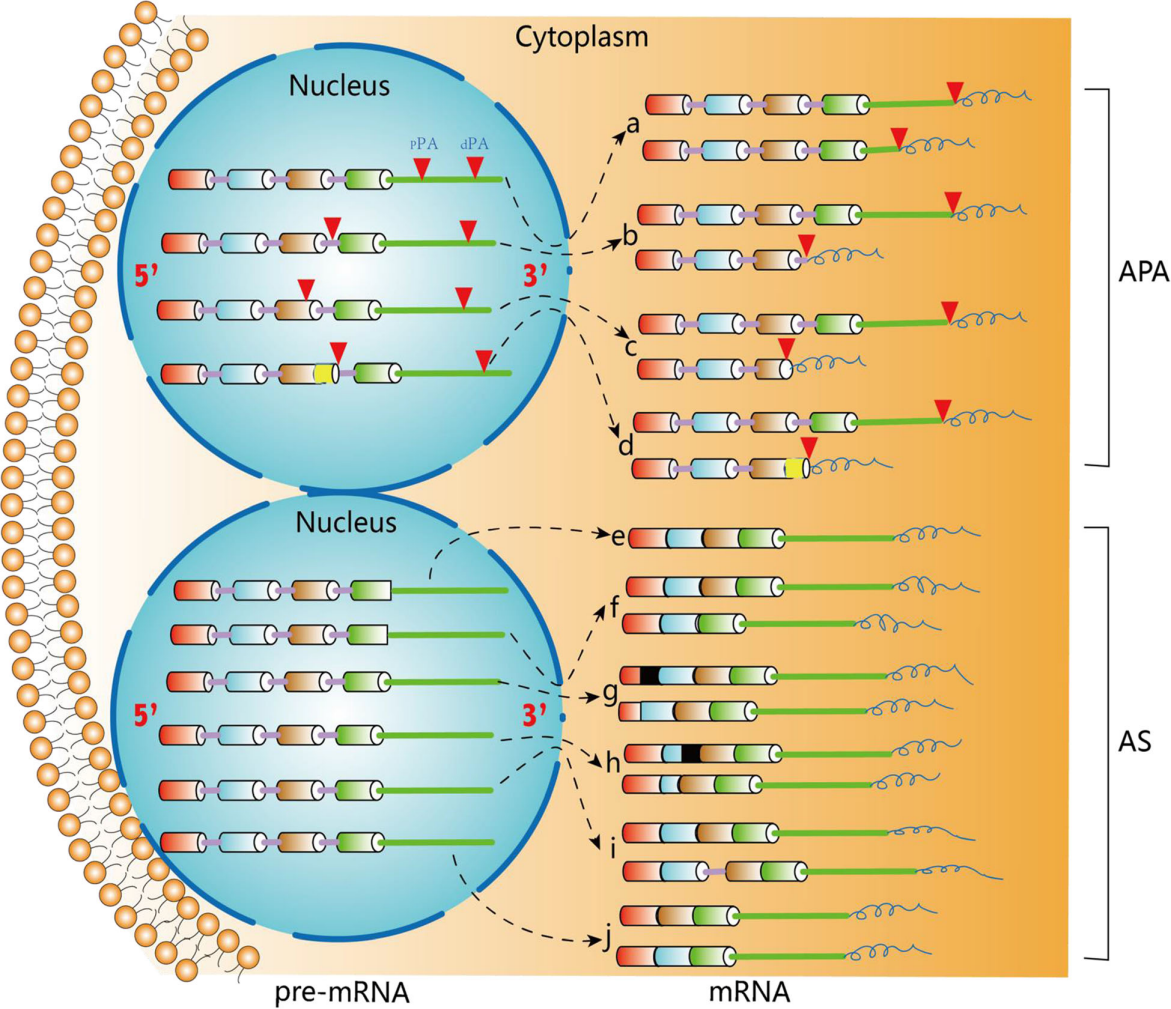
Comparison of APA and AS. **a**-**c** The patterns of APA can be classified into two main types: UTR-APA and CR-APA. For UTR-APA, alternative PAS resides in the 3′-UTR. Therefore, UTR-APA can generate transcripts with varying UTR lengths without changing the coding sequences. There are major types of CR-APA that may yield transcripts with truncated coding sequence. **d** Yellow is used to label the extended exon. For AS, e-constitutive splicing; **f** exon skipping/ inclusion;**g** alternative 5′-splice sites;**h** alternative 3′-splice sites; **i** intron retention; **j** mutually exclusive exons

Based on the locations of pAs, APA can be classified into two major categories: UTR-APA (Fig. 1a) and coding region-APA (CR-APA) (Fig. 1b-d). For CR-APA, alternative pAs are located in exons or introns. Therefore, CR-APA affects coding regions via alternative splicing (AS), leading to generation of protein isoforms with distinct C-termini [12, 13]. For UTR-APA, alternative pAs are located in the 3′-UTR, leading to the transcription products containing the same coding frame but variable 3′-UTRs. Previous studies suggested that global UTR-APA events are tissue-specific, with3′-UTR shortening positively correlates to cell proliferation and negatively to cellular differentiation [14–16].

The pre-mRNA 3′-processing complex is formed by several elements, including the canonical poly(A) signal sequence AAUAAA or its close variants (e.g. AAAUAA, AUAAAA, AUUAAA, AUAAAU, AUAAAG, CAAUAA, UAAUAA, AUAAAC, AAAAUA, AAAAAA, AAAAAG), which are utilized with varying frequencies throughout the genome, usually within 15–50 nts from the pA site [6, 8, 17–20]. UGUA elements are often located upstream of the pA site, U-rich elements are located near the pA site, and U/GU-rich elements are located within ~ 100 nts downstream of the pA site [7, 21, 22]. However, ~ 20% of human poly(A) signals are not surrounded by U−/GU-rich regions [23].

**Fig. 2:**
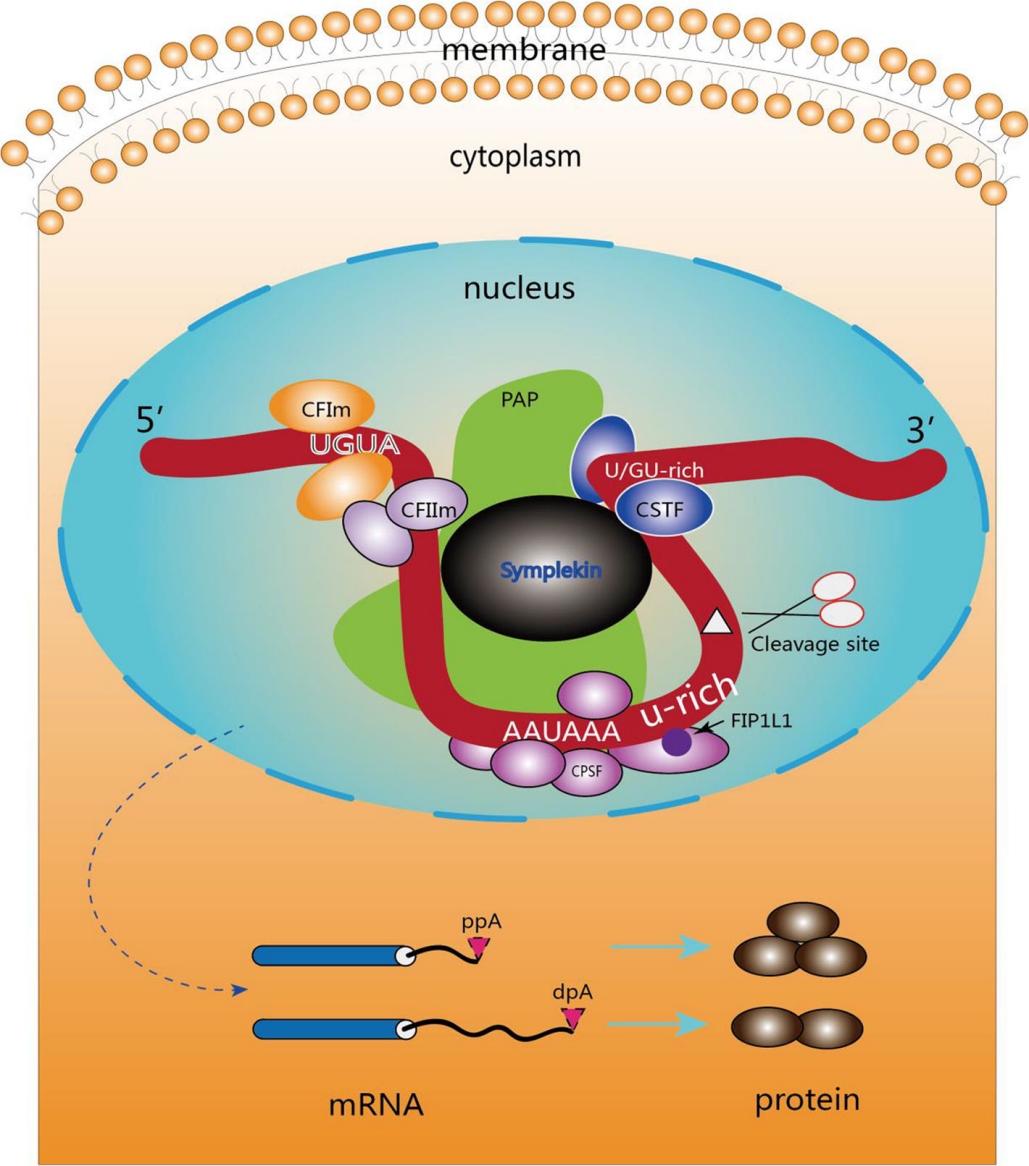
The APA complex and its machinery. CFIm complex binds to the conserved upstream UGUA region to mediate the cleavage reaction and recruit other proteins, including CPSF and CSTF. After combining with PAP, this complex translocates through the pre-mRNA in a 5′ to 3′ fashion. Upon arrival at the AAUAAA region, the adenosine acidification signal CPSF recognizes the polyadenylation signal AAUAAA and CPSF73 cleaves the mRNA. CSTF then binds to the GU- or U-rich sequence. The U-rich region bound to the FIP1L1 subunit of the CPSF is located between the polyadenylation signal AAUAAA and the cleavage site. Symplekin functions as a scaffold protein and PAPs catalyze the addition of untemplated adenosines. Generally, the usage of the proximal pAs generates short isoforms and the translation can be suppressed, often resulting in less protein

Out of 80 core factors in mammalian cells, about 20 of them are involved in the C/P machinery [24–26]. Generally, these core factors can be divided into four elements as followings (Fig. 2) [27, 28]:

**CPSF** (cleavage and polyadenylation specificity factor) is composed of CPSF1–CPSF4 (also known as CPSF160, CPSF100, CPSF73 and CPSF30), WDR33 and FIP1L1 (also known as Fip1) [22, 29]. The current understanding is that WDR33 and CPSF4 directly interact with pAs, and CPSF3 carries out the endonucleolytic cleavage [30, 31]. Working as a complex, CPSF recognizes the polyadenylation signal sequence AAUAAA and cleaves the pre-mRNA. This provides sequence specificity that may play an important role in regulating pA site selection, gene expression, cancer cell migration, metastasis, and eventually disease outcome [32]. As a part of CPSF complex, CPSF73 is an endonuclease that cleaves the pre-mRNA at the pA site [33]. However, under oxidative stress, CPSF73 translocates from the nucleus to the cytosol and causes significant inhibition of polyadenylation activity in prostate cancers [34]. Furthermore, Fip1, a member of the CPSF complex, potentially serves as a regulator of cellular self-renewal. Indeed, Fip1 depletion in mouse embryonic stem cells (ESCs) results in loss of cellular undifferentiated states and self-renewal capabilities due to the usage of preferred distal poly(A) site (dpA), ultimately leading to 3′-UTR lengthening of selected genes that determine the cell fate [35].

**CSTF** (cleavage stimulation factor) is composed of CSTF1, CSTF2, and CSTF3 (50 kDa, 64 kDa, and 77 kDa, respectively), and plays a key role in the cleavage reaction [36, 37]. CSTF complex can bind to the U- or GU-rich field downstream of the cleavage site to boost cleavage. For example, CSTF2, also known as CSTF64, directly interacts with the U/GU-rich region to modulate the 3′-terminal processing efficiency [38, 39]. Some studies reported that CSTF not only promote the usage of pAs, but also affect cell proliferation and potentially act as a biomarker of cancer invasion and prognosis [40, 41]. CSTF64 acts as an essential polyadenylation factor and a master regulator of 3′-UTR shortening across multiple tumor types. The expression of CSTF64 was found to be associated with poor lung cancer prognosis and overexpression of CSTF64 promoted lung cancer cell proliferation and invasion [25].

**CFI and CFII** (cleavage factors I and II) are consisted of CFIm25 (also known as NUDT21/nudix hydrolase 21/CPSF5), CFIm59 and CFIm68, all of which bind upstream of the conserved UGUA motif to mediate the cleavage reaction [28, 42]. CFIm binding can function as a primary determinant of pA sites by looping out an entire pA region and thereby inducing the selection of an APA site [43]. Other proteins, including symplekin, poly(A) polymerase (PAP), and poly(A) binding protein (PAB), can regulate APA site selection as well. PABs (PABII, RBBP6, PABPN1) bind to the growing poly(A) tail, preventing the interaction between CPSF and the poly(A) polymerase. Those activities primarily occur when the tail is ~ 250 nts and the purpose of which is to control poly(A) tail length while APA in progression [44, 45].

The factors involved in the C/P machinery usually participate in APA regulation. Among them, CFIm25 has been identified as the major global regulator of APA, whose knockdown not only induce a global switch to the use of proximal poly(A) signal, but also enhance target gene stability and expression [41, 46]. Huang et al. reported that CFIm25 depletion significantly increases the transcript levels of CCND1 and GSK3β, in addition to decrease the utilization of dPAS by several oncogenes (IGF1R, CCND1, and GSK3β) [46]. Furthermore, gene ontology analyses (GO) demonstrated that CFIm25 not only modulate APA via MAPK signaling pathways, but is also linked with cancer-associated signaling and protein ubiquitination signaling pathways [47]. Moreover, depletion of CFIm25 and CFIm68, but not CFIm59, leads to proximal polyadenylation site selection in HEK293 cells [48, 49]. However, Xia et al. reported that there are no CFIm25 expression differences between tumor tissue and healthy tissue [3]. Kubo et al. also reported that CFIm may not have a role for poly(A) site selection [27]. Additionally, Takagaki et al. demonstrated that CSTF64 is the first factor in APA 3′-end processing and that IgM can employ APA to activate mouse B-cells [50]. While it appears that CFIm plays a key role in the regulation of APA, its exact role still remains unclear [51].

RNA-binding proteins (RBPs) can also affect APA’s capability to target mRNAs by competing with or enhancing the binding of polyadenylation machinery proteins to their target sites [8]. Xiang et al. analyzed the global APA profiles from a large database across different cancer types and suggested that PABPN1 is the master regulator of APA profiling across different cancer types. A CTRP dataset demonstrated that PABPN1 expression is statistically correlated with the sensitivity towards 31 drugs [52]. RBPs can work alone to prevent the binding of other APA factors to the proximal poly(A) sites or affect APA selection through its role in maintaining RNA stability [53–55]. Furthermore, RBPs can regulate the dynamic APA profile and promote mitosis-to-meiosis transition [4].

### How APA is regulated

APA is a very comprehensive molecular biological process, involving numerous cellular elements. Currently, we still don’t know much about this unique biologic process. However, the situation has been rapidly improved in a very short period of time after the scientific community sensed the importance of APA in cellular biology and its potential role as a novel cancer therapy target. APA is a dynamically and spatiotemporally coordinated process of numerous core factors. For example, CFIm can bind to the specific RNA sequence in a pre-mRNA and then recruits the core factor CPSF through its interaction with a CPSF subunit, hFip15 [56]. CSTF-64 may interact with CPSF73, but not CFIm25. It was observed that both CSTF64 and CPSF73 levels are elevated in the cells that migrate into the healthy tissue, but not for CFIm25 level [17]. CFIm is involved in the early step of pre-mRNA 3’-processing complexe assembling via alternatively stimulating or suppressing cleavage and poly(A) addition depending on the levels of its own or other core factors, and the RNA sequence surrounding the potential cleavage sites [57].

Besides the core factors, a variety of physiological conditions also participate in APA regulation, such as the local chromatin structure, nucleosome positioning, DNA methylation, and histone modifications [58]. Interestingly, some factors participating in the 5′-terminal capping can also influence the efficiencies of both cleavage and polyadenylation [59].

Additionally, APA can be regulated at the transcription level. The transcription machinery, such as transcription initiation, progression, and splicing, is likely to affect the efficiency and specificity of polyadenylation [60]. Therefore, investigating the association between the specific sequence elements at the promoter region and the poly(A) site selection will greatly aid us in uncovering the mechanism behind this interesting phenomenon, which may potentially help in developing a novel cancer therapy strategy [61].

### How APA is methodologically analyzed

Since the effects of pAs in IgM and dihydrofolate reductase (DHFR) gene encoding were observed in 1980, a series of stringent research methods and strategies have been developed to identify and study APA, such as the *Poly(A)-ClickSeq* next-generation sequencing (NGS) technology [62–65]. With the support of these novel methodologies, especially with the advancement of NGS technology and the rapid accumulation of sequencing data from those gene expression variants, the experimentally determined genetic pA databases are continuously expanding [66, 67].

Based on 3′-enriched RNA-seq protocols, APA analysis methods can be classified mainly into two categories: oligo (dT) priming-based methods and RNA manipulation-based methods [5, 62, 68, 69]. Because the only reads mapped to the 3′ -termini of the mRNA are useful for APA discovery, the number of reads limited these methods. If the read coverage to 5′- and 3′-termini are low, RNA-seq will not be suitable for identifying pAs precisely and extensively. Moreover, another challenge is to resolve the read mapping ambiguity due to isoform transcripts overlap [18, 70]. Though it has reading length limitation, a range of RNA-seq algorithms have been developed to quantify relative changes in 3′-UTR length, therefore to predict APA events. Several pA detection and APA analytical methods and algorithms also have been developed in the last several years, such as Dynamic Analyses of Alternative PolyA Adenylation (DaPars), 3USS, MISO, Roar, QAPA, and Change Points [3, 71, 72] . A 2019 review by Gruber and Zavolaneloquently compared these methods [73] .

**DaPars** is the most popular data analysis method among them, although QAPA is more efficient and sensitive [74]. DaPars identifies distal pAs based on RNA-seq data, and then uses a regression model to perform de novo identification and quantification of dynamic APA events between two conditions, regardless of any prior APA annotation [3]. The probability of yielding sequenced reads is unified among individual isoforms. The pAs present at positions along gene locations that exhibit a distinct drop in RNA-seq read coverage [75]. After correcting the potential RNA-seq non-uniformity bias along the gene body, the exact location of proximal APA site can be identified, and the statistically significant dynamic APAs and their activities then will be detected. The key methodological innovation of DaPars is the direct inference of de novo APA events from existing RNA-seq data without relying on any additional experiments. Another advantage of DaPars is that it can resolve the overlapping of neighboring genes that may give false-positive results by increasing the cutoffs. However, due to non-uniform read coverage along loci, this method limits the accuracy of de novo poly(A) site detection by increasing the false positive rate.

**QAPA** quantitatively infers APA from conventional RNA-seq data by directly estimating the absolute alternative 3′-UTR isoform expression. It then computes the relative expression of each isoform among all isoforms to assess APA [74]. The limitation of QAPA is that it requires pre-defined pAs. However, this problem can be mitigated by the generation of an expanded resource of annotated pAs that incorporate data from 3′-UTR RNA-seq and other resources [74]. Because of reading coverage biases at the 3′-terminus of transcripts, poor yields of non-templated poly(A) tail-containing reads, and ambiguity of read mapping in overlapping transcript isoforms, the methods based on canonical RNA-seq data are limited while attempting to precisely map the pAs [18, 76]. However, with the advance of molecular technology, the methods to study APA have been continuously growing. Wang et al. used CRISPR/Cas9 methodology to study the biological function of APA via editing the weak poly(A) signal to a canonical poly (A) signal and directing the signals to target specific poly(A) sites [77].

In brief, each of current available APA analytic methods has its advantages and limitations. The analytical strategies based on canonical RNA-seq data are utilized most within the APA research community.

**Single-cell level study** The advantage of single-cell approach is that it can significantly reduce the background noise from bulk cells that contain a mixture of RNA material extracted from cells originating from various tissues or differentiations.

With the development of single-cell analysis technology, APA variations among the cells has been recently investigated [78]. Though single-cell APA research has rarely been conducted on a large scale, this technique works on high-depth and full-length of single-cell RNA-seq (scRNA-seq), which makes it a possible tool to accurately analyze APA. Jingle Bells and scRNA-SeqDB (https://bioinfo.uth.edu/scrnaseqdb/) utilized scRNA-seq datasets to investigate a variety of cancer types [79]. Ye et al. reported the use of scRNA-seq data to investigate dynamic APA usage variations in different bone marrow mononuclear cell types from a large sample collections containing both healthy controls and AML patients. They found that, in comparing to healthy individuals, AML patients appear to have lower APA diversity among eight different cell types. They further revealed extensive involvement of APA regulation in erythropoiesis during leukemia progression at the single-cell level [50]. By analyzing 515 scRNA-seq datasets extracted from 11 breast cancer patients, Kim et al. reported that cell-type-specific APA can be identified in single cell level based on 3′-UTR length variation in combination with gene expression level and APA patterns. Moreover, they demonstrated that immune-specific APA signatures in breast cancer can potentially be utilized as a prognostic marker for early stage breast cancers [31].

**APA and alternative splicing:** Though there are significant differences between APA and alternative splicing (AS), both APA and AS can generate various isoforms, even interacting with each other during pre-mRNA process. Additionally, while APA has four typical isoforms, AS has six (Fig. 2). Several in-depth analyses of transcriptomic data from various human tissues and cell lines revealed a strong correlation between APA and AS [6, 55, 80]. If the pA is within the terminal exon, the APA can act like a special type of AS, named CR-APA, which cannot possess an in-frame stop codon or 3′-UTR and is likely to be degraded rapidly through the non-stop code mediated mRNA decay process (Fig. 1b) [11, 12, 81]. Shen et al. reported that APA and splicing factor SRSF3 worked together to modulate the cell-aging process [82]. While APA may play a role in some splicing factor-mediated AS, splicing factors may also work with APA elements to assist in this process. For example, U2AF2 and RBPs are capable of interacting and recruiting CFI to facilitate 3′-terminus formation near the polypyrimidine tracts [79, 83]. Furthermore, CPSF complex can interact with splicing factor TFIID (transcription factor II D) in regulating RNA polymerase II [84, 85]. It is also observed that U1 snRNP (small nuclear ribonucleoprotein) can work within introns by suppressing premature cleavage and polyadenylation. U1 depletion also leads to the activation of intron poly(A) signals and causes genome-wide APA [86, 87].

AS and APA also compete each other while in CR-APA. For example, the ablation of the splicing factor 3B subunit1 (a component of U2 snRNP, also named SF3b1) can activate the intron PAS. U1 snRNP can also independently influence APA splicing activities [88]. Since U1 snRNP can bind to the 5′-terminal region of the transcript and block potential cleavage factor recognition, U1 snRNP knockdown increases the utilization of the pA sites within introns close to that transcript area [89, 90]. However, Movassat et al. demonstrated that the association between APA and AS is limited to terminal introns [91]. They also demonstrated that CstF64 knockdown can indirectly influence the AS of hnRNP A2/B1, but not APA, in HeLa cells [92].

### How APA regulate cell cycle

**Fig. 3:**
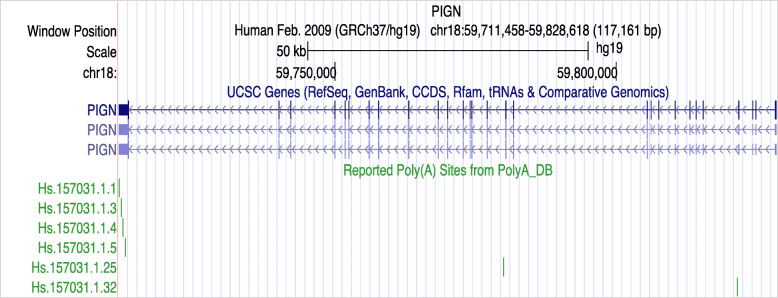
Track of PIGN by Grch37/hg19. PIGN location in chromosome18(q21.33) has three transcripts. There are 6 pAs in the polyA database

There are many genes, including *TP53*, *CDC6 (*cell division cycle 6), *CyclinD1* (CCND1), and *CDK* (cyclin-dependent kinase), are associated with cell cycle checkpoints and regulate cell cycle progression. As pre-mRNA usually has more than one pA sites, the cell cycle relevant gene products are modulated by the APA mechanism and generate various isomers. 3′-UTR shortening of *CDC6,* a major regulator of DNA replication, is linked to higher CDC6 protein levels and increased S-phase entry in breast cancer cells [93]. *Cyclin D1*, which plays a critical role in promoting G1–S phase transition in many cell types, is subject to APA regulation via both UTR-APA and CR-APA mechanisms [77, 94]. In addition, Xiang et al. examined the top 10% of all 20,532 genes associated with APA events and observed that most of these genes participate in chromatin structure-related activities, suggesting a relationship between APA processing and chromatin structure modification [53]. Mitra et al. found that APA acts as a linkage between cell cycle and tissue migration through analyzing mice dermal excisional wounds [17]. They demonstrated that proliferating cells adjacent to wounds express higher levels of APA factors than quiescent fibroblasts in unwounded skin. *PIGN,* which regulates cell cycle through interacting with the spindle assembly checkpoint proteins, is found to harbrt 6 pA sites in its 3′-UTR (Fig. 3) [95].

### How APA interacts with miRNA in post-transcriptional modulation

More than 50% of conserved microRNAs (miRNAs) target sites residing downstream of proximal pAs in mammalian genes. As a result, the UTR-APA plays a key role in regulating the interaction between transcripts and miRNAs [96]. APA is recently identified as a widespread mechanism controlling gene stability and expression. The miRNA targeting sites are mostly located in 3′-UTR [26, 52, 97]. The transcripts with shorter 3′-UTR lengths are usually more stable due to the loss of targeting sites for miRNAs. It was previously demonstrated that APA is a crucial regulatory mechanism in several cancer types, such as glioblastoma tumor, hepatocellular carcinoma, prostate cancer, and breast cancer [1]. However, Gruber et al. reported that 3′-UTR shortening has only a limited impact on murine and human T-lymphocyte proliferation. It also showed that not every APA event relates to higher protein levels [98]. Several studies have reported that the effects of APA on mRNA stability and ribosome loading are marginal, depending on the cell-type-specific miRNA expression and availability of RNA-binding proteins [37, 96]. A typical example is *PAX3* gene expression regulation. *PAX3* is a major regulator of myogenic differentiation, whose transcript has a miR-206 target site in the 3′-UTR. However, *PAX3* isoforms show variant differentiation patterns in different muscle types [98, 99].

APA can also modulate miRNA targets that are located in introns. The *ZFR* gene is targeted by its intronic miRNA (miR-579) in U87 cell line. Hinske et al. also reported that the APA signal plays a role in delivering miRNA negative feedback to *ZFP* gene [100].

APA affects gene expression not only by shortening the 3′-UTR to remove the miRNA targeting sites, but also via other molecular mechanisms. Masamha et al. reported that CFIm25 and miR-23 were independent in suppressing the expression of one of the glutaminase isoforms’ 3′-UTRs [22]. Therefore, although mRNA escapes miRNA suppression via shortening 3′-UTR to remove miRNA target site (a canonical APA mechanism), other APA and miRNA interaction mechanisms also coexist.

### Prospects

**Fig. 4:**
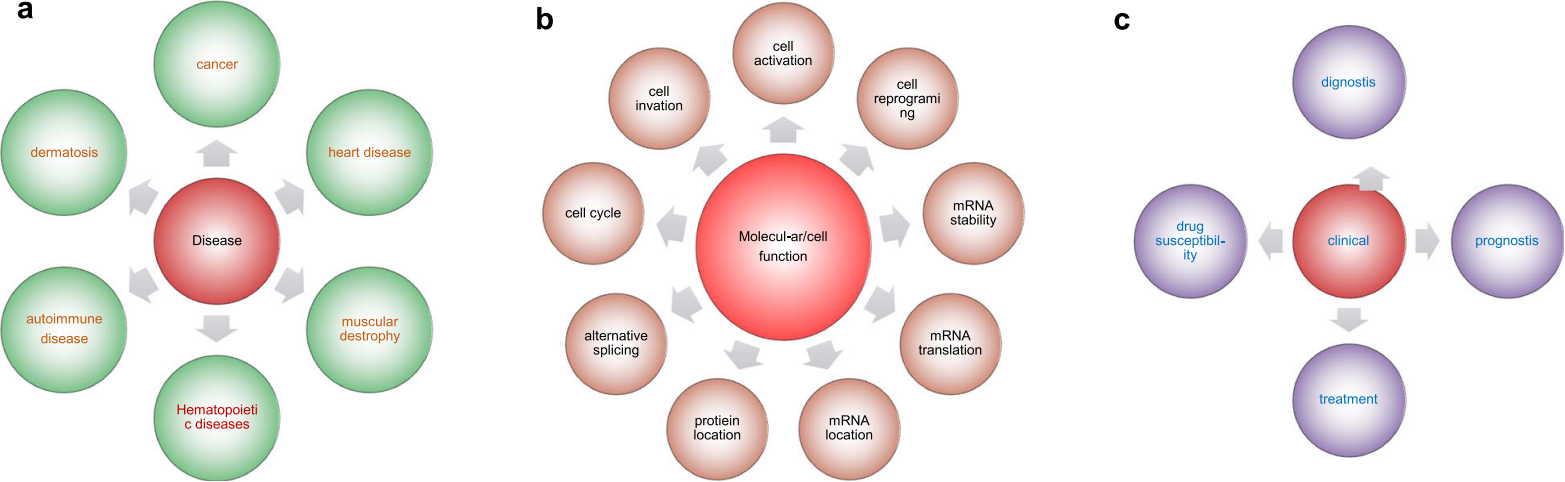
APA Impacts at molecular, cellular, and clinical levels. **a** APA can affect cell functions through various molecular mechanisms;**b**&**c** APA has relationship with many type diseases and the diagnosis, prognosis and treatment of diseases

APA is relatively a new biomedical research field. Though we achieved some milestone accomplishments on APA research in the last several years, much remains to be elucidated (Fig. 4). The APA studies have been focusing on the direct actions of various *trans-*acting factors in the last several years. Future investigations hopefully will concentrate on the signal regulation of those *trans-*acting factors at molecular and cellular levels. It is known that APA plays crucial roles in editing pre-mRNAs and determining the specificity and stability of the subsequent mRNA isoforms. APA participates in modulating innate antiviral immune response, regulating cancer initiation and prognosis, and developing drug resistance. Meanwhile, APA behaves differently on individual gene, cell type, tissue type, and even disease. Understanding APA and its comprehensive regulatory mechanisms in human diseases will open a new venue for pursuing precision medicine and personalized medicine.

## Conclusion

APA is a crucial post-transcriptional regulatory mechanism that could generate various mRNA isoforms from a single gene. Each mRNA isoform eventually translates into a protein product with unique biological functions. APA could regulate almost every major step of molecular cell biological process, such as cellular genomic stability, proliferation capability, and transformation feasibility. Our journal of understanding APA is just beginning. However, the emerging evidences indicate that APA, at least, could be potentially a biomarker for disease diagnosis, severity stratification, and prognostic forecast; and possibly a novel therapy target.

## Data Availability

Yes.

## Abbreviation

APA: Alternative polyadenylation
NGS: Next generation-sequencing
CFI/IIm: Cleavage factors I /II
CFIm25: Homolog of CFIM-25
CPSF: Cleavage and polyadenylation specificity factor
CSTF: Cleavage stimulation factor
RBPs: RNA-binding proteins
pAs: Cleavage/polyadenylation(C/P) sites
PABPN1: Polyadenylation binding protein 1
CR-APA: Coding region-APA
AS: Alternative splicing
ESCs: Embryonic stem cells
PAP: Poly(A) polymerase
PAB: Poly(A) binding protein
GO: Gene ontology analyses
DHFR: Dihydrofolate reductase
DaPars: Dynamic Analyses of Alternative PolyAAdenylation
3USS: A web server for detecting alternative 3′UTRs from RNA-seq experiments
MISO: mixture-of-isoforms model
Roar: detecting alternative polyadenylation with standard mRNA sequencing libraries
QAPA: Quantification of APA
scRNA-seq: Single-cell RNA-seq
BMMCs: Bone marrow mononuclear cells
TFIID: Transcription factor II D
SF3b1: Splicing factor 3B subunit1
*CDC6*: Division cycle 6
*CDK*: Cyclin-depentkinase
*PIGN*: Phosphatidylinositol glycan anchor biosynthesis class N
*PAX3*: Paired box 3 gene
Pre-mRNA: Pre-messenger RNA
